# Single Liver Lobe Repopulation with Wildtype Hepatocytes Using Regional Hepatic Irradiation Cures Jaundice in Gunn Rats

**DOI:** 10.1371/journal.pone.0046775

**Published:** 2012-10-16

**Authors:** Hongchao Zhou, Xinyuan Dong, Rafi Kabarriti, Yong Chen, Yesim Avsar, Xia Wang, Jianqiang Ding, Laibin Liu, Ira J. Fox, Jayanta Roy-Chowdhury, Namita Roy-Chowdhury, Chandan Guha

**Affiliations:** 1 Departments of Radiation Oncology and Pathology, Albert Einstein College of Medicine and Montefiore Medical Center, Bronx, New York, United States of America; 2 Departments of Medicine and Genetics, Albert Einstein College of Medicine, Bronx, New York, United States of America; 3 Department of Surgery, University of Pittsburgh School of Medicine, Children's Hospital of Pittsburgh of UPMC and McGowan Institute of Regenerative Medicine, Pittsburgh, Pennsylvania, United States of America; University of Birmingham, United Kingdom

## Abstract

**Background and Aims:**

Preparative hepatic irradiation (HIR), together with mitotic stimulation of hepatocytes, permits extensive hepatic repopulation by transplanted hepatocytes in rats and mice. However, whole liver HIR is associated with radiation-induced liver disease (RILD), which limits its potential therapeutic application. In clinical experience, restricting HIR to a fraction of the liver reduces the susceptibility to RILD. Here we test the hypothesis that repopulation of selected liver lobes by regional HIR should be sufficient to correct some inherited metabolic disorders.

**Methods:**

Hepatocytes (10^7^) isolated from wildtype F344 rats or Wistar-RHA rats were engrafted into the livers of congeneic dipeptidylpeptidase IV deficient (DPPIV^−^) rats or uridinediphosphoglucuronateglucuronosyltransferase-1A1-deficient jaundiced Gunn rats respectively by intrasplenic injection 24 hr after HIR (50 Gy) targeted to the median lobe, or median plus left liver lobes. An adenovector expressing hepatocyte growth factor (10^11^ particles) was injected intravenously 24 hr after transplantation.

**Results:**

Three months after hepatocyte transplantation in DPPIV^−^ rats, 30–60% of the recipient hepatocytes were replaced by donor cells in the irradiated lobe, but not in the nonirradiated lobes. In Gunn rats receiving median lobe HIR, serum bilirubin declined from pretreatment levels of 5.17±0.78 mg/dl to 0.96±0.30 mg/dl in 8 weeks and remained at this level throughout the 16 week observation period. A similar effect was observed in the group, receiving median plus left lobe irradiation.

**Conclusions:**

As little as 20% repopulation of 30% of the liver volume was sufficient to correct hyperbilirubinemia in Gunn rats, highlighting the potential of regiospecific HIR in hepatocyte transplantation-based therapy of inherited metabolic liver diseases.

## Introduction

Hepatocyte transplantation has been used as an alternative to whole liver transplantation for amelioration of metabolic deficiencies in a number of patients with various inherited liver-based metabolic disorders [1]–[7]. While these studies have demonstrated the safety and feasibility of this procedure, the number of hepatocytes that could be engrafted in single procedures was insufficient for complete cure of these diseases [8]–[11]. In the majority of inherited metabolic diseases of the liver, the longevity of the mutant hepatocytes is not significantly limited. Because the liver to body weight ratio is tightly regulated by physiological mechanisms, simple metabolic stimulation does not lead to extensive proliferation of the donor cells. Inhibition of the mitotic potential of the host cells provides a competitive proliferative advantage to the donor cells [12], [13], allowing massive proliferation of the donor cells, which replace significant fractions of the host hepatocyte mass in response to mitotic stimuli. Since the chemical methods used by many investigators for this purpose are probably not suitable for translation to clinical application, we have explored hepatic irradiation (HIR) to inhibit the growth of the host hepatocytes [14], [15]. Our results in rodent and non-human primate models show that preparative whole liver HIR augments the initial engraftment of the donor cells by transiently disrupting the endothelial barrier [11]. In addition, HIR allows the engrafted cells to respond more efficiently to mitotic stimuli, such as partial hepatectomy or expression of hepatic growth factor (HGF), than the host hepatocytes. This leads to extensive repopulation of the liver with complete correction of the metabolic defect in the uridinediphosphoglucuronate glucuronosyltransferase 1A1 (ugt1a1)-deficient Gunn rat model of Crigler-Najjar syndrome type 1 (CN1) [16] and a mouse model of primary hyperoxaluria type 1 (PH1) [17].

Despite the encouraging results in the rodent models, whole liver HIR has not been tested as a preparative regimen in clinic trials because the risk of RILD may be greater in primates, including humans. However, recent clinical studies using conformal HIR have shown that partial irradiation of the liver markedly reduces the occurrence of RILD and allows far greater doses of radiation to be delivered safely [18], [19]. Therefore, we wanted to test the hypothesis that preparative HIR of one or two selected liver lobes should result in a sufficient wild-type donor cell mass for correction of an inherited metabolic liver disease.

Initially, for the convenience of identification of the donor cells, we transplanted hepatocytes from F344 rats into congeneic DPPIV^−^ recipients by injection into the splenic pulp [15]. To verify the regiospecificity of liver repopulation, we irradiated one half of the median lobe, followed by mitotic stimulation by injecting an adenovector expressing HGF (Ad-HGF) intravenously [20]. To determine whether repopulation of one or two of the five liver lobes is sufficient to correct hyperbilirubinemia in Gunn rats, wildtype hepatocytes from congeneic Wistar rats (Roman High Avoidance, RHA strain) were transplanted after regional HIR to the median lobe (30% of liver mass) or median plus left anterior lobes (60% of liver mass). Mitotic stimulation was provided by injection of Ad-HGF. Our results demonstrate for the first time that repopulating a single liver lobe by regional HIR is sufficient to cure hyperbilirubinemia in Gunn rats. Combined with mitotic stimulation, conformal HIR permits marked liver repopulation by transplanted hepatocytes, thereby correcting hyperbilirubinemia, as seen after whole liver HIR.

## Materials and Methods

### Animals

Male, 250–300 g wildtype Fisher 344 (F344) rats were purchased from Taconic Farms (German Town, NY). Wildtype Wistar-RHA rats, congeneic Gunn rats and DPPIV- deficient F344 rats were bred and maintained on normal rat chow diet and the climate-controlled facility of the Institute for Animal Studies at the Albert Einstein College of Medicine in a 12-hr light/dark cycle. This study was approved by the Institutional Animial Care and Use Committee of the Albert Einstein College of Medicine.

### Viral Vector

An adenoviral vector expressing human HGF was generated by the Cell Culture and Genetic Engineering Core of the Marion Bessin Liver Research Center of the Albert Einstein College of Medicine [21].

### Regional Hepatic irradiation

Regional HIR in rats was performed as reported with some modifications [15]. Briefly, the liver was exposed by midline incision and all liver lobes and other abdominal organs were covered by 2 mm-thick lead shields, except the region targeted to be irradiated. We have previously conducted a dose escalation (15–50 Gy) study of preparative HIR and demonstrated 50 Gy was optimal in achieving >80% repopulation of Gunn rat livers by transplanted hepatocytes [16]. We, therefore, administered a HIR dose of 50 Gy selectively to the exposed half of the median lobe in DPPIV^−^ rats (n = 3), and the whole median lobe (n = 6) or median and left lobe (n = 8) in Gunn rats, using a Philips orthovoltage unit operating at 320 kVP, 5 mA, and 0.5 mm copper filtration at 200cGy/min. Control Gunn rats (n = 4) underwent laparotomy, but no HIR was delivered. In our study we had to deliver intra-operative HIR to rat livers in order to spatially confine the irradiation dose to specific liver lobes because of the use of orthovoltage animal irradiator and the small volume of liver in rats. However clinical translation of our preparative HIR technique could be easily achieved in humans via external beam RT using modern 3D conformal techniques. Indeed, fractionated external beam RT has been used as a preparative regimen for hepatocyte transplantation in F344 rats using a clinical linear accelerator [22], [23].

### Hepatocyte isolation and transplantation

Donor hepatocytes were isolated from DPPIV^+^ wildtype F344 rats or ugt1a1^+^ wildtype Wistar-RHA rats by an *in situ* two-step collagenase perfusion procedure [24]. Male DPPIV^−^ Fisher rats and Gunn rats (180–200 g body weight) that had received regional HIR served as recipients for congeneic normal F344 and Wistar-RHA rat hepatocytes, respectively. Twenty-four hours after conformal HIR, 10^7^ hepatocytes were transplanted by intrasplenic injection as described [16]. Ad-HGF (10^11^ particles) was injected via the tail vein 24 hours after hepatocyte transplantation [20]. Control rats received sham surgery (no HIR), Ad-HGF injection and hepatocyte transplantation.

### Histochemical staining for DPPIV activity

The median lobe subjected to conformal HIR was separated from other liver lobes and snap-frozen in pre-cooled 2-methylbutane at −80°C. Five-micron thick cryostat liver sections were fixed for 5 min in cold 95% ethanol/5% glacial acetic acid (99∶1vol/vol) on ice and followed by a 5 min wash in cold 95% ethanol on ice. The sections were then air-dried and stained for DPPIV histochemistry as previously described [25].

### Immunofluorescent staining for ugt1a1 expression

Five-micron frozen sections of liver tissues were fixed with ice-cold acetone/methanol for 45 minutes. Then the sections were incubated for 60 minutes at room temperature with the primary antibody (rabbit polyclonal antibody raised against a 31-mer polypeptide corresponding to the unique amino-terminal domain of rat ugt1a1) at 1∶50 dilution after blocking with donkey serum. The sections were washed thrice with phosphate-buffered saline (PBS) and incubated for 45 minutes at room temperature with the secondary antibody, Cy3-conjugated donkey anti-rabbit IgG (H+L) at 1∶200 dilution (Jackson ImmunoResearch Laboratories, Inc.). After extensive washing, the cover slips were mounted onto the slides with Vectashield (Vector Laboratories) and imaged using a Nikon Eclipse TE 2000-S fluorescent microscope.

### Measurement of ugt1a1 protein content in different lobes

Liver fragments (100–150 mg) from each lobe were homogenized in a glass-Teflon motorized homogenizer and protein concentrations were determined (Bio-Rad protein assay). The proteins (100 µg/lane) were subjected to electrophoresis on 5%–7.5% polyacrylamide gradient SDS/glycine gel. Following electrophoresis, proteins were transferred onto a polyvinylidene difluoride membranes (Bio-Rad). The membranes were blocked with 5% fat-free milk powder in PBS followed by incubation with the rabbit antiserum directed against the rat ugt1a1 amino-terminal domain. After washing with PBS, the membranes were incubated with a horseradish peroxidase–conjugated goat anti-rabbit IgG (Sigma) and developed using an enhanced chemiluminescence reagent (ECL Plus; Amersham, Arlington Heights, IL).

### Measurement of serum bilirubin levels

Approximately 0.4 ml of blood was collected from the rat tail vein before any experimental procedure (pretransplant) and at various time points after transplantation under isoflurane anesthesia, and serum bilirubin levels were determined using an automated clinical microchemistry system (Bayer Chem One; Bayer Corp., Tarrytown, NY).

### Analysis of bilirubin species excreted in bile

Bile samples were collected from the bile ducts of the Gunn rats after completion of serial blood collection, and were analyzed by reverse-phase high-performance liquid chromatography as described [16].

### Assay for ugt1a1 activity toward bilirubin

Ugt1a1 enzyme activity in the rat livers was analyzed as described previously [26].

### Statistical analysis

Repopulation of donor cells was assessed by counting the number of ugt1a1-stained donor hepatocytes in host liver lobes after ugt1a1 immunohistochemistry. Sampling regions were chosen at random for digital acquisition for data quantitation in a blinded fashion. A total of 20 fields at 200× magnification, each from irradiated and non-irradiated liver lobes of three rats per treatment group were used for each data point. The serum bilirubin values were expressed as the mean ± standard deviation (SD) and the ugt1a1 enzyme activity was expressed as percent of ugt1a1 activity in the wildtype Wistar RHA rat liver in order to estimate the extent of repopulation of host liver by donor hepatocytes. Statistical differences between two groups were determined by a two-sided Student *t* test. P values less than 0.05 were considered statistically significant.

## Results

### Targeted HIR enabled regiospecific liver repopulation in DPPIV^−^ recipients

**Figure 1:**
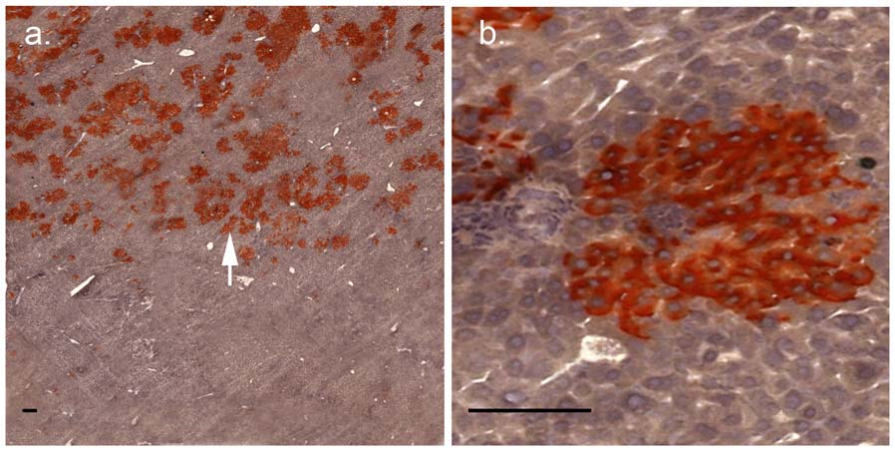
Regiospecific liver repopulation in DPPIV^−^ recipients by donor DPPIV^+^ hepatocytes following conformal HIR of one half of the median lobe. All liver lobes, except one half of the median lobe were shielded from HIR. Three months after hepatocyte transplantation, liver sections were stained histochemically for DPPIV activity. A region at the junction of the irradiated and non-irradiated regions of the median lobe is shown. Panel a. Low power view showing clusters of DPPIV-positive donor hepatocytes (red, white arrow) in the upper part of the section that had received preparative HIR, and the absence of such clusters in the lower part of the section that had been shielded from HIR. Panel b. High power view showing a donor hepatocyte cluster. The black bars indicate 100 µm.

To determine the effect of targeted HIR to the liver, DPPIV^+^ F344 hepatocytes were transplanted into congeneic DPPIV^−^ rat livers that had been subjected to preparative HIR of one half of the median lobe only. Three months after transplantation, 30–60% of the irradiated region of the median lobe was selectively replaced by DPPIV+ donor hepatocytes, indicating massive proliferation of the donor cells. In contrast, the non-irradiated half of the lobe contained only individual DPPIV^+^ donor hepatocytes, indicating lack of significant donor cell proliferation (Figure 1).

### Serum bilirubin levels in Gunn rat recipients

**Figure 2:**
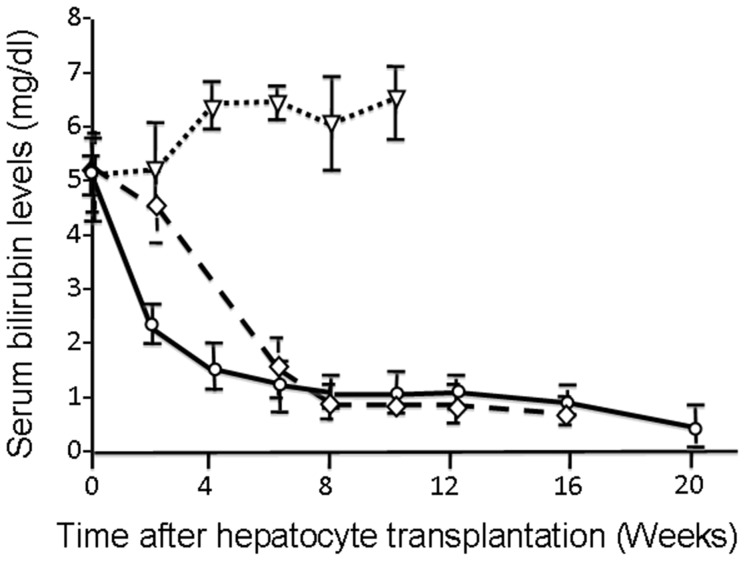
Conformal HIR followed by hepatocyte transplantation ameliorates bilirubin levels in a hyperbilirubinemia model. The control group of Gunn rats received Ad-HGF followed by hepatocyte transplantation without HIR (triangles, n = 4). One experimental group received Ad-HGF and preparative median lobe HIR (1/3 of liver, diamonds, n = 6) before hepatocyte transplantation. Another experimental group received Ad-HGF and preparative HIR of the median plus left lobe (2/3 of liver, circles, n = 8) before hepatocytes transplantation.

In order to demonstrate the functionality of the repopulated donor hepatocytes in irradiated host liver, we transplanted congeneic ugt1a1-proficient Wistar hepatocytes in jaundiced ugt1a1-deficient Gunn rats, following regiospecific preparative HIR. The effect of liver replacement in Gunn rat recipients by transplanted hepatocytes is shown in Figure 2. In the group receiving HIR of the median lobe (30% of the liver mass) only, mean serum bilirubin levels declined from pretransplantation values of 5.17±0.78 mg/dl to 0.96±0.30 mg/dl (mean ± SD) in 8 weeks after hepatocyte transplantation (p<0.0001), and remained close to that level throughout the study. In the group of Gunn rats that received median and left lobes irradiation (60% of the liver mass), serum bilirubin levels declined more rapidly than in the group receiving HIR of the median lobe only, so that the mean values were significantly lower in this group at 4-weeks. However, serum bilirubin concentrations in both groups stabilized at similar levels (1.12±0.22 mg/dl) in 8 weeks. In both HIR groups, the serum bilirubin levels were significantly lower than in the group receiving laparotomy and Ad-HGF, but no HIR, at all time points beyond 4 weeks after transplantation (p<0.0001).

### Analysis of bilirubin species excreted in bile

**Figure 3:**
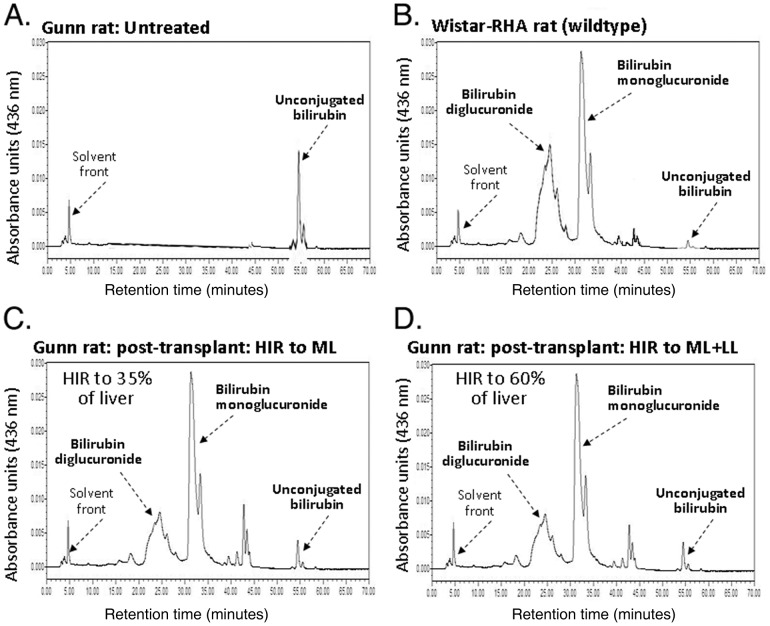
HPLC profile of bilirubin species excreted in bile. (A) The bile of untreated Gunn rats contained some unconjugated bilirubin, but no bilirubin glucuronides. (B) Bile of the wildtype Wistar-RHA donors contained predominantly bilirubin diglucuronide and monoglucuronide and a very small amount of unconjugated bilirubin. (C) Three months after hepatocyte transplantation following HIR to 1/3 of liver, the pigment profile of bile in transplant-recipient Gunn rats became similar to that of the normal Wistar rats, except that there was still a significant amount of unconjugated bilirubin in the bile. (D) Three months after hepatocyte transplantation following HIR to 2/3 of liver, the pigment profile of bile in transplant-recipient Gunn rats became similar to that of Gunn rats received HIR to 1/3 of liver.

Bile duct cannulae were placed, as previously described [16], at the end of the observation period in all Gunn rat recipients in order to examine the bilirubin species in the bile of recipient animals. Representative HPLC analyses of pigments excreted in the bile collected from untreated Gunn rats (Fig. 3A), congeneic wildtype Wistar-RHA rats (Fig. 3B) and Gunn rats receiving the various treatments are shown in Figure 3. As expected, the bile of untreated Gunn rats contained only unconjugated bilirubin, no bilirubin glucuronides (Figure 3A), whereas bile of the Wistar-RHA rats contained predominantly bilirubin diglucuronide and monoglucuronide, and only a small amount of unconjugated bilirubin (Figure 3B). In the groups receiving hepatocyte transplantation after preparative HIR targeted to the median lobe (Figure 3C) or the median plus left lobe (Figure 3D) of the liver, the pigment profile of bile became similar to that of the wildtype Wistar-RHA donor rats, except that there was still a significant amount of unconjugated bilirubin in the recipient Gunn rat bile.

### Immunofluorescent staining for ugt1a1 in different liver lobes

**Figure 4:**
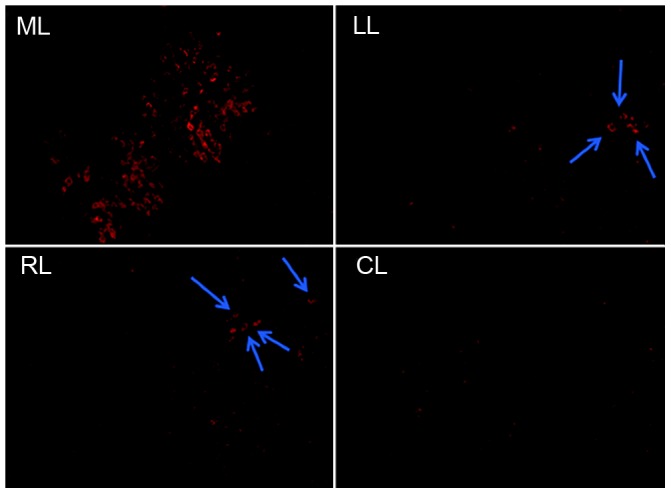
Immunofluorescent staining for ugt1a1 in different liver lobes. Three months after hepatocyte transplantation following HIR to median lobe (ML), 20% of the irradiated median lobe was replaced by donor hepatocytes, whereas the unirradiated left (LL), right (RL) and caudate (CL) lobes contained only small ugt1a1+ hepatocyte clusters.

Liver repopulation was examined three months after hepatocyte transplantation and conformal HIR to the median liver lobe, by immunofluorescent staining for ugt1a1. As expected, transplanted hepatocytes selectively repopulated the median lobe. Based on examination of 20 fields at 200× magnification, approximately 20% (range 18–22%) of the irradiated lobe was replaced by ugt1a1^+^ donor hepatocytes, whereas the unirradiated left, and right lobes contained only small occasional ugt1a1^+^ hepatocyte clusters. When both the median and the left lobe were irradiated, 18–30% hepatocytes in each lobe were repopulated by the transplanted cells. The caudate lobe, which receives the least scattered radiation because of its anatomical location, contained only individual engrafted donor hepatocytes (Figure 4).

### Ugt1a1 content of the different lobes

**Figure 5:**
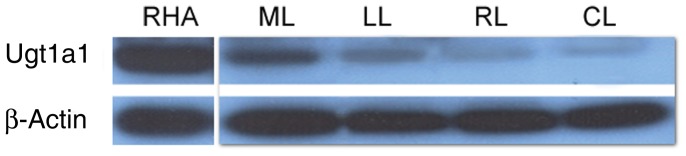
Western blot for ugt1a1 in the Gunn rat liver following HIR to 2/3 of liver and hepatocyte transplantation. Three months after hepatocyte transplantation, ugt1a1 protein was demonstrated in the irradiated and unirradiated liver lobes of Gunn rats by western blot analysis. However, the ugt1a1 content in irradiated lobes was much higher than that of unirradiated lobes. RHA, Wistar-RHA rat liver; ML, median lobe; LL, left lobe;, RL, right lobe; CL, caudate lobe.

Three months after hepatocyte transplantation and preparative HIR to the median and left lobe, 52-kd ugt1a1-immunoreactive bands were observed in all four lobes of Gunn rat livers. However, the ugt1a1 content in the irradiated median and left lobe was much higher than that of unirradiated right and caudate lobe (Figure 5).

### Ugt1a1 activity toward bilirubin

**Figure 6:**
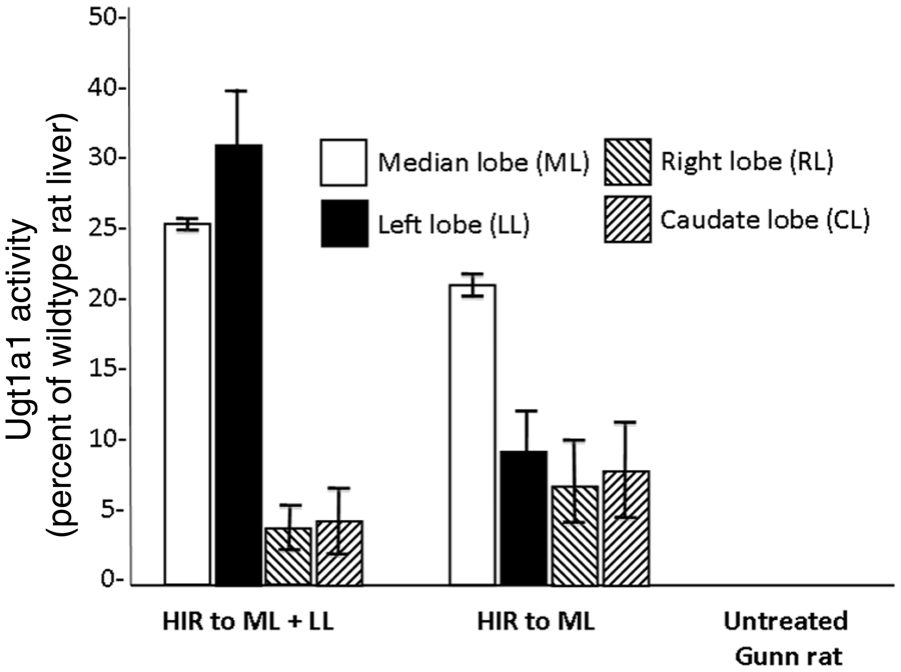
Ugt1a1 activity toward bilirubin in Gunn rats that received conformal HIR before hepatocyte transplantation. Ugt1a1 activities in liver homogenates of different recipient groups as in Figure 2 are shown (means±SD) as percent of the activity in the liver of Wistar-RHA rats (2.6 µmol/g liver per hour).

Following transplantation, ugt1a1 activity in the liver homogenates was assayed with bilirubin as the acceptor substrate. In Gunn rat livers that received regiospecific HIR prior to hepatocyte transplantation, ugt1a1 activity in irradiated median and left lobes was about 25–40% of the activity found in control Wistar-RHA rat livers (2.6±0.14 µmol/g liver per hour). These values were significantly higher than in the unirradiated lobes of Gunn rat livers, which were less than 5% of the activity in livers of Wistar-RHA rats (P<0.01). Similarly, ugt1a1 activity in the median lobe of Gunn rats that had received preparative HIR to that lobe only was about 21–24% of the activity in wildtype RHA rats, and was significantly higher than the activity in the unirradiated left, right, and caudate lobes. The ugt1a1 activity was undetectable in untreated Gunn rats (Figure 6).

## Discussion

Crigler-Najjar syndrome type 1 (CN1) is a potentially lethal autosomal recessive disorder characterized by life long non-hemolytic unconjugated hyperbilirubinemia. The disorder results from a deficiency of ugt1a1 activity, which is critical for glucuronidation and excretion of bilirubin [27]. Although phototherapy and plasmapheresis treatment have extended the life expectancy of patients with this disorder, liver transplantation is still the only definitive treatment [27]. As liver transplantation is a formidable surgical procedure, hepatocyte transplantation is being explored as a minimally invasive alternative to whole organ transplantation in the management of several liver-based metabolic disorders and liver failure [1]–[7], [27]. Because a fraction of the normal hepatic enzyme content is often sufficient to support metabolic function, we and others have proposed that transplantation of isolated hepatocytes might cure several inherited liver-based metabolic disorders. After extensive preclinical research in animal models, we performed hepatocyte transplantation in a patient with CN1, which resulted in a significant reduction of serum bilirubin levels [1]. However, the levels were not low enough to obviate the need for phototherapy. Following our report, several groups confirmed that hepatocyte transplantation successfully, but partially, ameliorated hyperbilirubinemia in CN1 patients [1], [4], [28], [29]. Thus, it was clear that the number of hepatocytes that could be safely engrafted into the liver of CN1 patients was not sufficient to achieve complete cure of the disease.

We then performed further preclinical studies to achieve proliferation of the engrafted hepatocytes within the liver. Host hepatocytes in CN1 patients, as in most other liver-based inherited metabolic disorders, have normal proliferative capacity. As the liver to body weight ratio is maintained within a tight limit by strong physiological mechanisms, the engrafted wildtype cells can proliferate only by replacing the host hepatocytes. Massive hepatic repopulation has been achieved by genetic, chemical and physical means to limit the mitotic capacity of the host [12], [14], [30], [31]. Under these circumstances, mitotic stimuli after hepatocyte transplantation can cause massive preferential proliferation of the host cells, which competitively repopulate the host liver [13].

As the chemical methods used to inhibit host hepatocyte proliferation are not readily translatable to clinical use, we investigated preparative HIR for achieving liver repopulation by transplanted hepatocytes. These studies showed that whole liver HIR in combination with a mitotic stimulus, such as partial hepatectomy or HGF expression, results in massive liver repopulation and complete correction of the metabolic defect in Gunn rats [16], as well as in a mouse model of PH1 (hepatic alanine∶glyoxylate aminotransferase deficiency) [17], [20]. In addition, we have found that preparative HIR enhances engraftment in non-human primates [11]. A major hurdle to the application of this promising technology to clinical hepatocyte transplantation is concern about inducing RILD. Whole liver irradiation in primates, including humans, is associated with RILD that differs in severity and pathological characteristics from HIR-induced RILD in rodents. Therefore, it is critical to determine the tolerable dose of HIR in humans, and, if possible devise strategies to prevent clinical RILD. In humans, irradiation targeted to parts of the liver is tolerated much better than whole liver irradiation. It has been reported that therapeutic irradiation of one third of the liver volume in humans is tolerated up to doses as high as 90 Gy without manifestation of RILD [18], [19]. In fact, when radiation was targeted to <25% of the liver volume, RILD did not occur at any irradiation dose tested.

In contrast to the treatment of the liver by drugs or chemicals, modern conformal irradiation technology permits HIR delivery to precisely targeted regions. This should permit repopulation of specific regions of the liver, keeping the remaining liver unperturbed. To take advantage of this unique characteristic of irradiation, it would be important to estimate the volume of the liver that must be repopulated to achieve a cure of a metabolic liver disease. Our results in the DPPIV^−^ recipient rats demonstrate that the area of the liver to be repopulated can be precisely mapped by targeting preparative HIR to that area. Percent repopulation of the liver was somewhat lower in the Gunn rat recipients than in the DPPIV- rats, which could have resulted from the strain difference. Nevertheless, we show that as little as 20% repopulation of 30% of the liver volume in the Gunn rat recipients was sufficient to correct hyperbilirubinemia. Repopulation of twice that volume reduced serum bilirubin levels more rapidly, but ultimately the long-term effect was similar with both HIR groups. Excretion of bilirubin glucuronides in the bile confirmed the function of the repopulating donor cells in vivo. In addition, there was a significant unconjugated bilirubin peak in the bile of the transplant recipients. A similar pattern is also found in human subjects with Gilbert syndrome, who have a partial deficiency of bilirubin glucuronidation [32]. This finding is consistent with the fact that the ugt1A1 deficiency was partially, but not completely normalized. In terms of potential clinical application, it should be noted that the therapeutic objective in patients with CN1 is to reduce serum bilirubin concentrations to safe levels, which can be clearly achieved by partial restitution of hepatic bilirubin glucuronidation.

Although we used an adenoviral vector to express HGF, for clinical application the mitotic stimulation will need to be achieved by alternative methods. Humanized activating antibodies to c-met, which have a much longer half-life in the circulation are being evaluated toward this goal in preclinical studies.

In conclusion, we have shown for the first time that targeted irradiation of liver lobes can be used to precisely delineate a fraction of the liver volume to be repopulated by transplanted hepatocytes. Partial repopulation of one third of the liver volume using this method cured hyperbilirubinemia in the Gunn rat model of CN1. Our finding is highly relevant to potential clinical application of preparative HIR for hepatocyte transplantation because irradiation of a limited liver volume is tolerated much better than whole liver irradiation in the clinic. Modern conformal irradiation techniques can precisely delineate regions of the liver to be irradiated, thereby avoiding the development of RILD. In combination with newer approaches to providing mitotic stimulation to the engrafted hepatocytes, regiospecific HIR could pave the way for a safe and highly effective hepatocyte transplantation-mediated treatment regimen for a large number of liver-based inherited metabolic diseases.

## References

[1] Fox IJ, Chowdhury JR, Kaufman SS, Goertzen TC, Chowdhury NR, et al (1998) Treatment of the Crigler-Najjar syndrome type I with hepatocyte transplantation [see comments]. N Engl J Med 338: 1422–1426. 9580649 10.1056/NEJM199805143382004

[2] Lake JR (1998) Hepatocyte transplantation [editorial; comment]. N Engl J Med 338: 1463–1465. 9580657 10.1056/NEJM199805143382012

[3] Dhawan A, Strom SC, Sokal E, Fox IJ (2010) Human hepatocyte transplantation. Methods Mol Biol 640: 525–534. 20645072 10.1007/978-1-60761-688-7_29

[4] Fisher RA, Strom SC (2006) Human hepatocyte transplantation: worldwide results. Transplantation 82: 441–449. 16926585 10.1097/01.tp.0000231689.44266.ac

[5] Horslen SP, McCowan TC, Goertzen TC, Warkentin PI, Cai HB, et al (2003) Isolated hepatocyte transplantation in an infant with a severe urea cycle disorder. Pediatrics 111: 1262–1267. 12777539 10.1542/peds.111.6.1262

[6] Legido-Quigley C, Cloarec O, Parker DA, Murphy GM, Holmes E, et al (2009) First example of hepatocyte transplantation to alleviate ornithine transcarbamylase deficiency, monitored by NMR-based metabonomics. Bioanalysis 1: 1527–1535. 21083101 10.4155/bio.09.112

[7] Waelzlein JH, Puppi J, Dhawan A (2009) Hepatocyte transplantation for correction of inborn errors of metabolism. Curr Opin Nephrol Hypertens 18: 481–488. 19770757 10.1097/MNH.0b013e3283318e1c

[8] Gupta S, Bhargava KK, Novikoff PM (1999) Mechanisms of cell engraftment during liver repopulation with hepatocyte transplantation. Semin Liver Dis 19: 15–26. 10349680 10.1055/s-2007-1007094

[9] Soltys KA, Soto-Gutierrez A, Nagaya M, Baskin KM, Deutsch M, et al (2010) Barriers to the successful treatment of liver disease by hepatocyte transplantation. J Hepatol 53: 769–774. 20667616 10.1016/j.jhep.2010.05.010 PMC2930077

[10] Gupta S, Rajvanshi P, Sokhi R, Slehria S, Yam A, et al (1999) Entry and integration of transplanted hepatocytes in rat liver plates occur by disruption of hepatic sinusoidal endothelium. Hepatology 29: 509–519. 9918929 10.1002/hep.510290213

[11] Yamanouchi K, Zhou H, Roy-Chowdhury N, Macaluso F, Liu L, et al (2008) Hepatic irradiation augments engraftment of donor cells following hepatocyte transplantation. Hepatology 49: 258–267. 10.1002/hep.22573 PMC3416044 19003915

[12] Grompe M, Laconi E, Shafritz DA (1999) Principles of therapeutic liver repopulation. Semin Liver Dis 19: 7–14. 10349679 10.1055/s-2007-1007093

[13] Oertel M, Menthena A, Dabeva MD, Shafritz DA (2006) Cell competition leads to a high level of normal liver reconstitution by transplanted fetal liver stem/progenitor cells. Gastroenterology 130: 507–520. 16472603 10.1053/j.gastro.2005.10.049

[14] Guha C, Parashar B, Deb NJ, Sharma A, Gorla GR, et al (2001) Liver irradiation: a potential preparative regimen for hepatocyte transplantation. Int J Radiat Oncol Biol Phys 49: 451–457. 11173140 10.1016/s0360-3016(00)01495-4

[15] Guha C, Sharma A, Gupta S, Alfieri A, Gorla GR, et al (1999) Amelioration of radiation-induced liver damage in partially hepatectomized rats by hepatocyte transplantation. Cancer Res 59: 5871–5874. 10606225

[16] Guha C, Parashar B, Deb NJ, Garg M, Gorla GR, et al (2002) Normal hepatocytes correct serum bilirubin after repopulation of Gunn rat liver subjected to irradiation/partial resection. Hepatology 36: 354–362. 12143043 10.1053/jhep.2002.34516

[17] Guha C, Yamanouchi K, Jiang J, Wang X, Roy Chowdhury N, et al (2005) Feasibility of hepatocyte transplantation-based therapies for primary hyperoxalurias. Am J Nephrol 25: 161–170. 15849463 10.1159/000085408

[18] Dawson LA, Normolle D, Balter JM, McGinn CJ, Lawrence TS, et al (2002) Analysis of radiation-induced liver disease using the Lyman NTCP model. Int J Radiat Oncol Biol Phys 53: 810–821. 12095546 10.1016/s0360-3016(02)02846-8

[19] Dawson LA, Ten Haken RK (2005) Partial volume tolerance of the liver to radiation. Semin Radiat Oncol 15: 279–283. 16183482 10.1016/j.semradonc.2005.04.005

[20] Jiang J, Salido EC, Guha C, Wang X, Moitra R, et al (2008) Correction of hyperoxaluria by liver repopulation with hepatocytes in a mouse model of primary hyperoxaluria type-1. Transplantation 85: 1253–1260. 18475180 10.1097/TP.0b013e31816de49e

[21] Wang X, Mani P, Sarkar DP, Roy-Chowdhury N, Roy-Chowdhury J (2009) Ex vivo gene transfer into hepatocytes. Methods Mol Biol 481: 117–140. 19096805 10.1007/978-1-59745-201-4_11

[22] Christiansen H, Koenig S, Krause P, Hermann RM, Rave-Frank M, et al (2006) External-beam radiotherapy as preparative regimen for hepatocyte transplantation after partial hepatectomy. Int J Radiat Oncol Biol Phys 65: 509–516. 16690433 10.1016/j.ijrobp.2006.01.040

[23] Krause P, Wolff HA, Rave-Frank M, Schmidberger H, Becker H, et al (2011) Fractionated external beam radiotherapy as a suitable preparative regimen for hepatocyte transplantation after partial hepatectomy. Int J Radiat Oncol Biol Phys 80: 1214–1219. 21514075 10.1016/j.ijrobp.2011.02.035

[24] Neufeld DS (1997) Isolation of rat hepatocytes.; Pollard JW, editor. Totowa, NJ: Humana Press Inc.

[25] Piazza GA, Callanan HM, Mowery J, Hixson DC (1989) Evidence for a role of dipeptidyl peptidase IV in fibronectin-mediated interactions of hepatocytes with extracellular matrix. Biochem J 262: 327–334. 2573346 10.1042/bj2620327 PMC1133264

[26] Roy-Chowdhury N, Roy-Chowdhury J, Wu G, Shouval R, Arias IM (1981) Bilirubin mono- and diglucuronide formation by human liver in vitro: assay by high-pressure liquid chromatography. Hepatology 1: 622–627. 6796486 10.1002/hep.1840010610

[27] Roy-Chowdhury N, Roy-Chowdhury J (2009) Disorders of bilirubin metabolism. In: Arias IM, editor. The Liver: Biology and Pathobiology. 5th ed. Chichester, West Sussex, UK ; Hoboken, NJ: John Wiley & Sons. pp. 251–256.

[28] Ambrosino G, Varotto S, Strom SC, Guariso G, Franchin E, et al (2005) Isolated hepatocyte transplantation for Crigler-Najjar syndrome type 1. Cell Transplant 14: 151–157. 15881424 10.3727/000000005783983250

[29] Dhawan A, Mitry RR, Hughes RD (2006) Hepatocyte transplantation for liver-based metabolic disorders. J Inherit Metab Dis 29: 431–435. 16763914 10.1007/s10545-006-0245-8

[30] Laconi E, Oren R, Mukhopadhyay DK, Hurston E, Laconi S, et al (1998) Long-term, near-total liver replacement by transplantation of isolated hepatocytes in rats treated with retrorsine. Am J Pathol 153: 319–329. 9665494 10.1016/S0002-9440(10)65574-5 PMC1852941

[31] Overturf K, Al-Dhalimy M, Tanguay R, Brantly M, Ou CN, et al (1996) Hepatocytes corrected by gene therapy are selected in vivo in a murine model of hereditary tyrosinaemia type I [see comments] [published erratum appears in Nat Genet 1996 Apr;12(4):458]. Nature Genetics 12: 266–273. 8589717 10.1038/ng0396-266

[32] Fevery J, Blanckaert N, Heirwegh KPM, Préaux A-M, Berthelot P (1977) Unconjugated Bilirubin and an increased proportion of bilirubin monoconjugates in the bile of patients with Gilbert's Syndrome and Crigler-Najjar Disease. J Clin Invest 60: 970–979. 409736 10.1172/JCI108877 PMC372448

